# Recognition of lipid A variants by the TLR4-MD-2 receptor complex

**DOI:** 10.3389/fcimb.2013.00003

**Published:** 2013-02-12

**Authors:** Nina Maeshima, Rachel C. Fernandez

**Affiliations:** Department of Microbiology and Immunology, University of British ColumbiaVancouver, BC, Canada

**Keywords:** TLR4, innate immunity, lipid A, signaling, LPS

## Abstract

Lipopolysaccharide (LPS) is a component of the outer membrane of almost all Gram-negative bacteria and consists of lipid A, core sugars, and O-antigen. LPS is recognized by Toll-like receptor 4 (TLR4) and MD-2 on host innate immune cells and can signal to activate the transcription factor NFκB, leading to the production of pro-inflammatory cytokines that initiate and shape the adaptive immune response. Most of what is known about how LPS is recognized by the TLR4-MD-2 receptor complex on animal cells has been studied using *Escherichia coli* lipid A, which is a strong agonist of TLR4 signaling. Recent work from several groups, including our own, has shown that several important pathogenic bacteria can modify their LPS or lipid A molecules in ways that significantly alter TLR4 signaling to NFκB. Thus, it has been hypothesized that expression of lipid A variants is one mechanism by which pathogens modulate or evade the host immune response. Additionally, several key differences in the amino acid sequences of human and mouse TLR4-MD-2 receptors have been shown to alter the ability to recognize these variations in lipid A, suggesting a host-specific effect on the immune response to these pathogens. In this review, we provide an overview of lipid A variants from several human pathogens, how the basic structure of lipid A is recognized by mouse and human TLR4-MD-2 receptor complexes, as well as how alteration of this pattern affects its recognition by TLR4 and impacts the downstream immune response.

## Innate immunity in the host

Innate immunity is the first line of defense against pathogens and involves the recognition of pathogen-associated molecular patterns (PAMPs), by pattern recognition receptors expressed by the host cell. This initial recognition is crucially important for the immediate innate immune response, and also for directing the later pathogen-specific adaptive immune response. While these molecular patterns are considered to be relatively conserved structures, there is in fact significant diversity within the basic structural framework, and the ability to modify PAMPs may be an important tool for pathogenic microorganisms to evade or modulate immune responses.

## LPS

**Figure 1:**
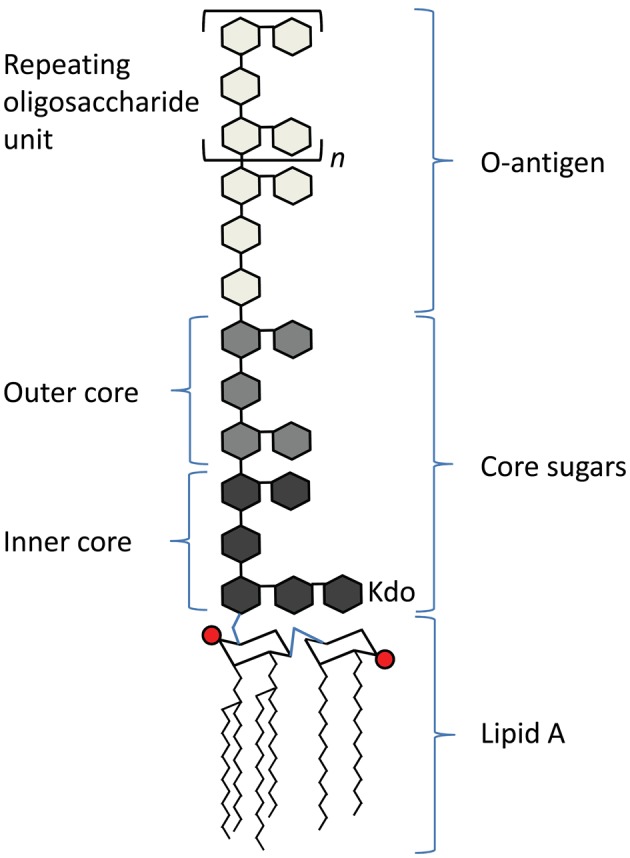
**Schematic of the basic structure of lipopolysaccharide.** LPS consists of three regions: from the bottom, lipid A (chair structure indicates di-glucosamine headgroup, red circles indicate phosphate groups, squiggly lines indicate acyl chains), core sugars, and O-antigen, which consists of repeating units (denoted in brackets, with an “n”) of oligosaccharides.

Lipopolysaccharide (LPS) is a well-characterized PAMP found in the outer leaflet of the outer membrane of most Gram-negative bacteria. It has three regions (Figure 1), a lipid A molecule (endotoxin), which anchors LPS in the outer membrane, a core sugar consisting of 3-deoxy-D-*manno*-oct-2-ulosonic acid (Kdo) moieties, and the O antigen, which consists of repeating oligosaccharide units (Raetz and Whitfield, 2002). The lipid A component of LPS is recognized by Toll-like receptor 4 (TLR4) and its co-receptor MD-2 on host cells (Kawai and Akira, 2010).

LPS molecules, due to their amphiphilic nature, can form aggregates in aqueous environments above a critical micellar concentration (Brandenburg and Seydel, 2010). The structure of these aggregates is influenced by the shape of the individual LPS molecules as well as environmental factors such as temperature or pH; furthermore, the type of aggregate structure formed can predict its level of biological activity (Brandenburg and Seydel, 2010). Lamellar aggregate structures, such as those formed by cylindrically-shaped LPS molecules, are associated with poor activation of TLR4, whereas cubic or hexagonal structures, such as those formed by conical molecules, are highly active (Erridge et al., 2002; Brandenburg and Seydel, 2010).

It is thought that monomeric LPS molecules are extracted from supramolecular structures prior to presentation to MD-2 and TLR4 (Gioannini et al., 2004), however it is still a point of debate whether the immunologically relevant unit of LPS, the form which is recognized by the host, is the monomer or the aggregate. Human pathogens have been shown to modify their LPS in ways that affect its shape, aggregate structure, and thus its ability to activate TLR4 (Zughaier et al., 2007). Although the response to LPS aggregates cannot be overlooked, here, we have focused on the interaction of monomeric LPS and TLR4.

## TLR4

TLR4 is a member of the Toll-like receptor family. There are 10 known human TLRs which recognize distinct PAMPs. TLR1, 2, and 6 combine to recognize bacterial lipoproteins, TLR4 recognizes LPS, and TLR5 recognizes flagella; these TLRs are expressed on the cell surface. TLR3, 7, 8, and 9 are found in endosomal compartments and recognize bacterial or viral nucleic acids [reviewed in (Kawai and Akira, 2011)]. TLRs are type I transmembrane proteins: they consist of an extracellular region made up of conserved leucine-rich repeat (LRR) domains, which recognize PAMPs, a transmembrane region, and the intracellular region, which contains Toll/IL-1 receptor (TIR) signaling domains needed to recruit adaptor proteins and facilitate signaling [reviewed in (Kang and Lee, 2011)].

## Signaling and functional consequences of TLR4 recognition of LPS

**Figure 2:**
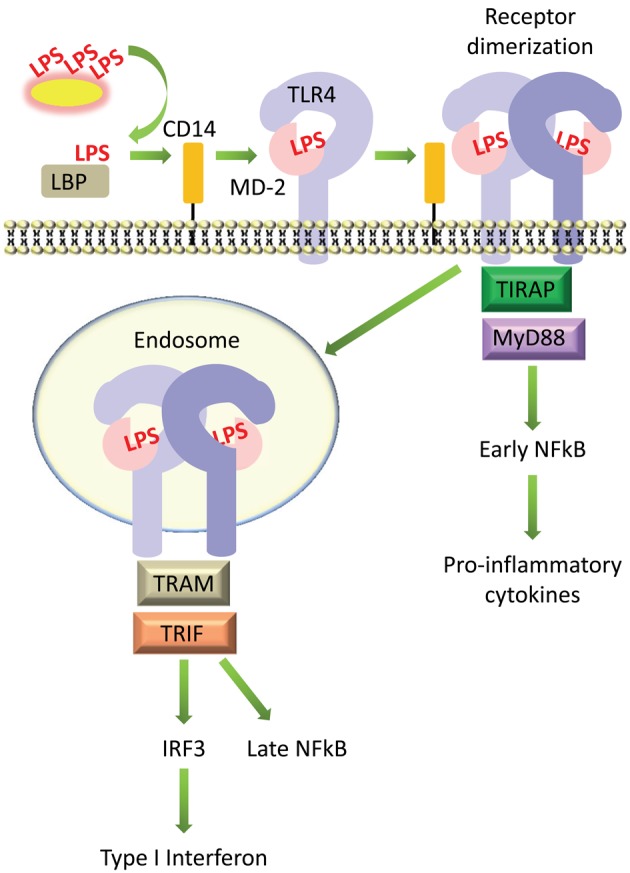
**Simplified diagram of signaling by LPS on host cells.** LPS is extracted from bacterial membranes by LPS binding protein (LBP) in serum, passed to CD14, then transferred to MD-2 and TLR4, which form a complex on the cell surface. LPS-induced receptor dimerization is followed by recruitment of the adaptor proteins TIRAP and MyD88, which promotes activation of the transcription factor NFκB. The TLR4-MD-2 complex can also be internalized, which recruits a different set of adaptor proteins, TRIF and TRAM, promoting activation of IRF3 and production of type I interferons, as well as delayed activation of NFκB.

LPS is extracted from bacterial membranes by LPS binding protein (LBP) in serum. LBP then transfers LPS to CD14, which can be found either in soluble form or linked to the cell surface by a glycosyl-phosphatidylinositol (GPI) anchor. CD14 presents LPS to the TLR4 and MD-2 (TLR4-MD-2) receptor complex. Upon binding LPS, the TLR4-MD-2 heterodimer is thought to dimerize, which brings together their intracellular TIR domains and creates a scaffold for the recruitment of adaptor proteins [reviewed in (Lu et al., 2008) and summarized in Figure 2].

TLR4 can then signal via two distinct pathways, the MyD88-dependent and TRIF-dependent pathways (Figure 2). In the MyD88-dependent pathway, dimerization of TLR4-MD-2 at the cell surface recruits the adaptor proteins TIRAP (MAL) and MyD88 (Kagan and Medzhitov, 2006). This initiates a signaling cascade that culminates in the activation of the transcription factor NFκB and the production of pro-inflammatory cytokines such as TNFα and IL-6 (Takeuchi and Akira, 2010). TLR4 is additionally internalized into endosomes where it recruits a different set of adaptor proteins, TRAM and TRIF, which signal to the transcription factor IRF3 and promote the production of type I interferons; this pathway also leads to “late” activation of NFκB (Kawai and Akira, 2011). The internalization of TLR4 into endosomes and activation of the TRIF pathway is facilitated by the activation of phosphatidylinositol-3-OH kinase [PI(3)K], specifically the p110δ isoform, which generates phosphatidylinositol-(3,4,5,)-trisphosphate, or PIP_3_, and leads to the dissociation of TIRAP from the plasma membrane, resulting in its degradation (Aksoy et al., 2012). Activation of the TRIF pathway via TLR4 internalization and production of type I interferons is controlled by CD14 (Jiang et al., 2005; Zanoni et al., 2011). Although the TRIF pathway is not always activated upon LPS stimulation, for example, in cells which do not express CD14 (Zanoni et al., 2011), it makes an essential contribution toward the stimulation of an appropriate immune response. Inhibition of the p110δ isoform of PI(3)K led to increased production of MyD88-dependent cytokines such as TNFα and IL-6, lower production of TRIF targets such as IFNβ and IL-10, and increased endotoxin-induced death (Aksoy et al., 2012).

Mice deficient in TLR4 have an increased susceptibility to infection with Gram-negative bacteria (O'Brien et al., 1980; Cross et al., 1989), but excessive activation of NFκB can lead to septic shock (Liu and Malik, 2006), and inappropriate TLR4 activation, for example, by nickel ligands (Schmidt et al., 2010) or house-dust-mite allergen Derp2, which is structurally and functionally similar to MD-2 (Trompette et al., 2009), can lead to allergy. Thus, the ability of TLR4 to recognize a variety of molecular patterns within a “conserved” LPS signature is important for host defense, but this same flexibility, if not controlled, leaves the host vulnerable to excessive or unwanted inflammatory responses. Work over the last two decades has revealed a complex interplay between the ability of pathogens to modulate their lipid A molecules, and the host's ability to recognize and differentially respond to these variant ligands.

## *E. coli* LPS has been used as a model for LPS recognition by TLR4 and MD-2

**Figure 3:**
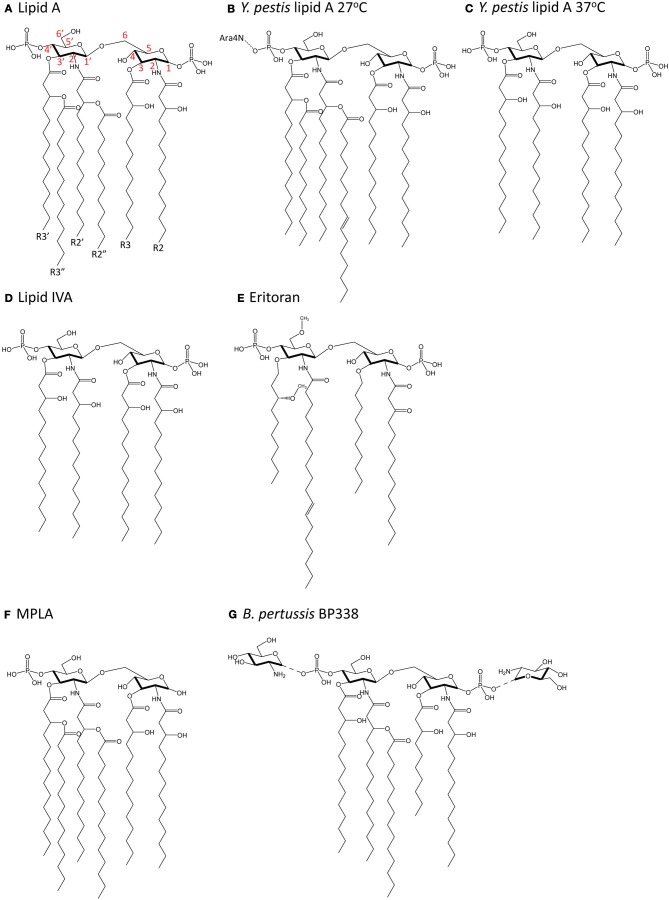
**Chemical structure of lipid A molecules. (A)** Hexa-acylated lipid A from *E. coli*. Red numbers indicate carbon numbering. **(B)** Hexa-acylated lipid A from *Y. pestis* grown at 27°C. **(C)** Tetra-acylated lipid A from *Y. pestis* grown at 37°C. **(D)** Tetra-acylated lipid IVA, a biosynthetic precursor of hexa-acylated lipid A. **(E)** The synthetic molecule Eritoran. **(F)** Monophosphoryl lipid A (MPLA). **(G)** Glucosamine-modified lipid A from *B. pertussis* (strain BP338, a Tohama I derivative).

Most of what is known about how lipid A in LPS is recognized by the TLR4-MD-2 complex on animal cells has been studied using hexa-acylated lipid A from *Escherichia coli*, which is a strong agonist of TLR4 signaling. *E. coli* lipid A (Figure 3A) consists of a di-glucosamine backbone, 1- and 4′-phosphate groups, and six fatty acyl chains, of which four are directly linked to the glucosamine head group at positions 2, 3, 2′ and 3′, and two are secondary chains attached to the hydroxyl groups of the 2′- and 3′-linked chains (R2″ and R3″, respectively). *E. coli* lipid A is synthesized in a series of reactions catalyzed by genes in the Raetz pathway. The first step is the acylation of UDP-N-acetylglucosamine (UDP-GlcNAc) at the C-3 position by LpxA. Next, LpxC catalyzes the deacetylation at C-2, followed by acylation of the C-2 amide by LpxD to yield UDP-2,3-diacylglucosamine. This is cleaved by the pyrophosphatase LpxH to release UMP and generate lipid X. Lipid X is then condensed with a second molecule of UDP-2-3-diacylglucosamine by LpxB, forming a disaccharide, diglucosamine-1-phosphate. LpxK then adds a phosphate group at the C-4′ position to yield lipid IVA. A Kdo sugar group is added at C-6′ by KdtA (or WaaA) and the final steps of lipid A biosynthesis involve the addition of lauroyl and myristoyl groups by LpxL and LpxM at positions C-2′ and C-3′, respectively, to yield lipid A [reviewed in (Raetz and Whitfield, 2002)].

**Figure 4:**
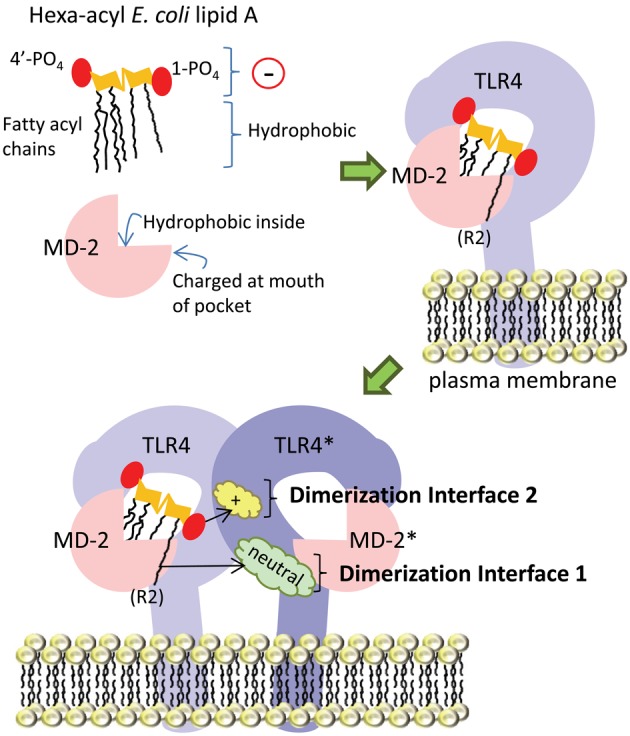
**Simplified diagram of TLR4-MD-2 receptor complex dimerization upon ligation of hexa-acyl lipid A.** The amphipathic lipid A molecule consists of negatively-charged (indicated by a circle with a “−” sign in it) phosphate groups and hydrophobic fatty acyl chains. The fatty acyl chains sit inside the “binding pocket” structure of the MD-2 co-receptor protein, which is lined with hydrophobic amino acid residues, while the phosphate groups are engaged by charged amino acids in MD-2 at the mouth of the pocket. In the next part of the figure, we show lipid A inside the MD-2 pocket and also in contact with TLR4, with which it forms a 1:1 complex. Upon lipid A binding, this TLR4-MD-2 complex dimerizes with a second complex. From this position, lipid A engages the TLR4 molecule in the second complex (TLR4^*^) at two main interfaces: the first is mediated by interaction of the 6th acyl chain of lipid A (which is shown sticking out of the pocket) with uncharged amino acids (cloud labeled “neutral”) on TLR4^*^, the second is mediated by interaction of the negatively-charged 1-phosphate (1-PO_4_) on lipid A with positively-charged amino acid residues (cloud labeled “+”) on TLR4^*^.

In the following section, the basic mechanism underlying *E. coli* hexa-acyl lipid A recognition by the host (human) TLR4-MD-2 receptor complex will be discussed. We have summarized this in simplified cartoon form in Figure 4. Briefly, the lipid A portion of LPS sits partially inside the co-receptor protein MD-2. Both lipid A and MD-2 interact directly with TLR4. Upon ligand (lipid A) binding, this complex of TLR4 and MD-2 dimerizes with a second TLR4-MD-2 complex, which facilitates signaling to downstream effectors. Although many amino acid residues in both TLR4 and MD-2 have been identified as contributing to this interaction, we have focused on two key interfaces where lipid A interacts with the second TLR4 molecule in the dimerized complex in order to discuss the potential significance of modification of the regions of lipid A found to interact at these interfaces.

## How does TLR4-MD-2 recognize lipid A?

TLR4 cannot recognize lipid A on its own, and requires MD-2 (Shimazu et al., 1999), with which it forms a stable 1:1 complex (Kim et al., 2007). MD-2 consists of two anti-parallel beta sheets which fold into a large open binding pocket lined with hydrophobic amino acid residues: this pocket accommodates the hydrophobic fatty acyl chains of lipid A (Figure 4). Furthermore, there are charged residues at the opening of the pocket which can interact with negatively-charged phosphate groups on the di-glucosamine backbone (Park et al., 2009). Thus, the amphipathic lipid A molecule is accommodated by hydrophobic and charged regions in the MD-2 binding pocket. The interface between the MD-2 co-receptor and TLR4 is made up of a network of hydrogen bonds; mutations in amino acid residues in MD-2 that interact with TLR4 (C95, C105, D99-101), as well as mutations in amino acid residues in TLR4 that interact with MD-2 (C29 and C40), disrupt this interaction (Re and Strominger, 2003; Nishitani et al., 2006).

In the dimerized TLR4-MD-2 complex, several key interactions at two defined interfaces have been identified (Figure 4). The crystal structure of human TLR4-MD-2 receptor with *E. coli* hexa-acyl LPS (Park et al., 2009) shows the formation of an m-shaped multimer made up of two molecules of TLR4 and two molecules of MD-2. *E. coli* lipid A is positioned in the hydrophobic pocket of MD-2 such that five of six acyl chains are completely buried inside the pocket, and the 6th chain (the R2 chain, as indicated in Figure 4) is partially exposed on the surface of MD-2 (Park et al., 2009). This is the first interface important for dimerization: the hydrophobic R2 chain forms hydrophobic interactions with F440^*^, F463^*^, and L444^*^ in the second TLR4 molecule (Park et al., 2009), hereafter referred to as TLR4^*^. In Figure 4, these amino acid residues would be found in the green cloud labeled “neutral.” The so-called “phenylalanine loop” of MD-2, around amino acid residue F126, undergoes a structural shift upon ligand binding. Thus, hydrophilic residues in the F126 loop, and R90 in MD-2, can form hydrogen bonds and ionic interactions with TLR4^*^: these surround and support the hydrophobic first dimerization interface (Park et al., 2009). The second dimerization interface consists of hydrophilic interactions between the 1-phosphate of lipid A and positively-charged residues in TLR4^*^, (the yellow cloud labeled with a “+” sign in Figure 4). In order for the acyl chains to fit inside the MD-2 binding pocket, the phosphorylated diglucosamine backbone of lipid A is displaced upwards by 5 Å, which allows the phosphate groups to interact with positively-charged lysine and arginine residues in TLR4, TLR4^*^, and MD-2, as well as by making a hydrogen bond to S118 of MD-2 (Park et al., 2009). Notably, this mechanism is relatively conserved between human and mouse TLR4-MD-2, both of which are potently activated by hexa-acyl LPS (Ohto et al., 2012a).

### Variations in lipid a affect its recognition

As lipid A is required for bacterial growth [*Neisseria meningitidis* being a notable exception (van der Ley and Steeghs, 2003)], its basic biosynthetic pathway is highly conserved among Gram-negative bacteria (Raetz et al., 2007). Mutations in any of the genes catalyzing the first seven steps in the Raetz pathway are lethal in *E. coli* (Trent et al., 2006). Bacteria can also express unique variants of this basic structure, which affect the host immune response, for example, by modulating resistance to host-derived cationic antimicrobial peptides (CAMPs), or by differentially affecting host TLR4 signaling (Trent et al., 2006). As an example, *Helicobacter pylori* is a widespread human pathogen that lives in the gastric mucosa (Taylor and Blaser, 1991). It expresses the canonical hexa-acyl lipid A structure only in minor amounts; the major species is mono-phosphorylated, decorated with a phosphoethanolamine group on the 1-phosphate, and has only three or four acyl chains (Moran et al., 1997; Suda et al., 1997, 2001). This structure of lipid A is much less stimulatory for TLR4 than hexa-acyl lipid A from *E. coli* (Cullen et al., 2011) and promotes resistance to the antimicrobial peptide polymyxin (Tran et al., 2006).

It is now appreciated that pathogenic bacteria have evolved numerous mechanisms for the modification of lipid A, including the expression of enzymes to add or remove acyl chains, phosphate groups, or catalyze the addition of chemical groups onto the phosphates. The ability of Gram-negative bacteria to respond to environmental signals by regulating the expression of lipid A modifying enzymes was initially described for the human pathogen *Salmonella enterica* serovar Typhimurium (*S*. Typhimurium); however, homologues for these enzymes, as well as the resulting lipid A variants, have been described in other human pathogens as well [reviewed in (Ernst et al., 2001; Miller et al., 2005)].

Here, we will summarize how variations in lipid A structure can affect its recognition by TLR4, broadly divided into modifications to the hydrophobic acyl chains at the first dimerization interface, and modifications to the charged phosphate groups at the second interface.

### Variations in lipid a acyl chains and their effect on the host immune response

Variation in the number of acyl chains in lipid A can impact signaling through TLR4 and dramatically alter the host immune response to the pathogen. LpxL and LpxM, as described earlier, catalyze the final two steps in the Raetz pathway, the addition of secondary acyl chains on lipid A. In *E. coli*, LpxL (also called HtrB) catalyzes the addition of the C-2′ lauroyl group on lipid A (Clementz et al., 1996). LpxL1 is a homologue of *E. coli* LpxL that is expressed in *N. meningitidis*, and catalyzes the addition of the C-2′ secondary lauroyl group (van der Ley et al., 2001). *N. meningitidis* expresses a hexa-acylated lipid A with secondary acyl chains at the C-2 and C-2′ positions (Kulshin et al., 1992); however, inactivating mutations in *lpxL1* have been found in meningococcal disease isolates of *N. meningitidis* (Fransen et al., 2009). *LpxL1* mutants express penta-acylated lipid A and have a decreased ability to stimulate the production of the pro-inflammatory cytokine TNFα in a human macrophage cell line (van der Ley et al., 2001). Furthermore, penta-acylated *N. meningitidis* lipid A has a decreased ability to induce human dendritic cell maturation (measured by upregulation of co-stimulatory molecules and production of cytokines such as TNFα, IL-12p70, and IL-10) compared to wildtype hexa-acylated lipid A (Zughaier et al., 2006; Steeghs et al., 2008), suggesting that inactivating mutations in *lpxL1* might represent an immune evasion strategy. Penta-acylated structures were found to bind to human MD-2 with lower affinity than hexa-acylated structures (Zimmer et al., 2007). However, while LPS from the *lpxL1* mutant also induced poorer NFκB activation in a reporter assay in HeLa cells, this effect was only seen in cells expressing human, but not mouse, TLR4 and MD-2 (Steeghs et al., 2008). In a similar vein, penta-acylated LPS from an *lpxL1* mutant could antagonize TNFα production induced by *E. coli* LPS in human peripheral blood mononuclear cells, but not in mouse macrophages (Sprong et al., 2011).

LpxM (also called MsbB), which is not essential for growth in *E. coli* (Karow and Georgopoulos, 1992), catalyzes the myristoylation of lipid A at the 3′ position and is important for generation of hexa-acyl lipid A (Clementz et al., 1997). *LpxM* mutants in *E. coli* produce predominantly penta-acylated lipid A (Clementz et al., 1997), which does not activate NFκB in reporter assays or induce TLR4 oligomerization *in vitro* (Tsuneyoshi et al., 2006). Similarly, stimulation of a macrophage cell line with purified LPS from *Yersinia pestis* strain KM218 lacking *lpxM* yielded reduced levels of the pro-inflammatory cytokine TNFα compared to wildtype (Feodorova et al., 2009). Mutants in *S. enterica* are poorer at stimulating production of TNFα and IL-1β in mouse dendritic cells at low multiplicities of infection (Kalupahana et al., 2003), and mutants in *Shigella flexneri* induces less inflammation in the intestine (D'Hauteville et al., 2002). Interestingly, the decreased ability to stimulate TLR4 makes *S. flexneri* less pathogenic (D'Hauteville et al., 2002); a similar trend was seen in *E. coli*, where *lpxM* mutation is also sufficient to make clinical isolates of *E. coli* less lethal in BALB/c mice (Kim et al., 2004).

Changes in acylation also affect TLR4 activation in *Y. pestis*, whereby temperature-dependent expression of tetra-acyl lipid A resulted in decreased stimulation of TLR4 (Kawahara et al., 2002). At 27°C, *Y. pestis* lipid A is hexa-acylated and modified by an aminoarabinose group on the phosphate group (Figure 3B). At 37°C (Figure 3C), *Y. pestis* expresses lipid A with only four acyl chains and no aminoarabinose modification (Kawahara et al., 2002). This occurs because the expression of the acyltransferase, *lpxP* is temperature-sensitive in *Y. pestis* (Rebeil et al., 2006). Tetra-acyl lipid A stimulated decreased production of the pro-inflammatory cytokine TNFα downstream of TLR4, and this effect was greater in human than mouse macrophages (Kawahara et al., 2002), pointing to a host-specific contribution to the response. Others have manufactured more strongly agonistic LPS in *Y. pestis* by expressing *lpxL* from *E. coli* (Montminy et al., 2006). This strain did not kill wildtype mice, but did kill TLR4- or MD-2-deficient mice, suggesting that TLR4 recognition of more immunostimulatory LPS was needed for the generation of a protective immune response (Montminy et al., 2006).

Recent work has shown that mice expressing human TLR4-MD-2, as opposed to mouse TLR4-MD-2, have severely decreased production of other pro-inflammatory cytokines, including IL-12p40 and IL-6, and are more susceptible to *Y. pestis* infection (Hajjar et al., 2012). Thus, at the body temperature of a human host, *Y. pestis* expresses a form of lipid A that allows it to evade the immune response in a species-dependent, TLR4-dependent manner.

Lipid A modifying enzymes which catalyze the addition or removal of acyl chains in response to environmental stimuli have also been described. PagL catalyzes the removal of an R-linked 3-hydroxymyristate from lipid A, and is under the control of the PhoP/PhoQ regulon in *S*. Typhimurium (Trent et al., 2001a). Deacylated lipid A from *E. coli* expressing PagL was found to induce significantly less NFκB activation in a reporter assay compared to unmodified lipid A (Kawasaki et al., 2004). *Bordetella pertussis* expresses a penta-acylated lipid A (Caroff et al., 2000). A mutant strain of *B. pertussis* was generated that expressed a homologue of *pagL* from *B. bronchiseptica*; the lipid A from this strain was found to consist mostly of tetra-acylated lipid A that was missing the C-3 position hydroxymyristate (Geurtsen et al., 2006). Stimulation of a human macrophage cell line with this strain resulted in significantly decreased production of the pro-inflammatory cytokine IL-6 (Geurtsen et al., 2006). Outer membrane vesicles from this mutant strain, compared to wildytpe, induced significantly lower expression of the cytokines IL-6 and IL-1β in the lung following intranasal challenge in mice (Asensio et al., 2011). Analysis of the bronchoalveolar lavage from infected mice showed a decrease in the proportion of Gr-1^+^ neutrophils, again suggesting a decrease in airway inflammation (Asensio et al., 2011).

PagP adds a palmitate to the 2 position N-linked hydroxymyristate of lipid A (Guo et al., 1998). In *S.* Typhimurium, it is also regulated by the PhoP/PhoQ system and is important for resistance to CAMPs (Guo et al., 1997). Expression of *S.* Typhimurium PagP in *E. coli* generates hepta-acyl lipid A, which had a reduced ability to activate NFκB in a luciferase reporter assay (Kawasaki et al., 2005b). A homologue of PagP has been described in *B. bronchiseptica*, where PagP expression is controlled by the Bvg virulence regulon. *B. bronchiseptica* in Bvg^+^ phase expresses a hexa-acylated lipid A, whereas a mutant strain lacking *pagP* expresses a penta-acylated lipid A (Preston et al., 2003). PagP is necessary for persistent colonization of the mouse respiratory tract (Preston et al., 2003) and resistance to serum-mediated killing (Pilione et al., 2004).

Palmitoylation of lipid A has also been described in *Pseudomonas aeruginosa* (Ernst et al., 1999), although a PagP homologue has not been found in *P. aeruginosa*, which suggests the expression of an alternate enzyme with this function. *P. aeruginosa* is an opportunistic pathogen, and has been known to cause chronic lung infections in cystic fibrosis (CF) patients (McIsaac et al., 2012). While a laboratory-adapted strain of *P. aeruginosa* was found to express penta-acylated lipid A, isolates from the airways of CF patients had hexa-acyl lipid A, due to the addition of a palmitate group (Ernst et al., 1999). Hexa-acylated LPS from CF isolates induced higher levels of IL-8 in HUVEC cells compared to penta-acylated LPS (Ernst et al., 1999). CF LPS also induced increased production of the pro-inflammatory cytokine TNFα in THP-1 cells and higher levels of NFκB activity in a reporter assay (Hajjar et al., 2002). Interestingly, these effects were species-specific: cells expressing mouse TLR4-MD-2 responded equally well to penta- and hexa-acylated structures (Hajjar et al., 2002).

LpxR is a 3′-O-deacylase which catalyzes the removal of the 3′ acyloxyacyl group from lipid A and was first characterized in *S*. Typhimurium (Reynolds et al., 2006). Mutants in *S*. Typhimurium where *lpxR* was deleted induced increased inducible nitric oxide synthase (iNOS) production by RAW264.7 macrophages, suggesting a negative effect of LpxR on TLR4 stimulation (Kawano et al., 2010). Orthologues of *lpxR* were additionally identified in several other organisms, including *H. pylori*, as well as *Y. enterocolitica*, where LpxR activity was again correlated with decreased TLR4 stimulation under certain conditions (Reines et al., 2012).

Recently, glycine or di-glycine modification of lipid A has been described in *Vibrio cholerae* (Hankins et al., 2012). The presence of the glycine modification did not significantly impact TLR4 activation, but was important for polymyxin resistance, perhaps by reducing the negative charge of the bacterial surface (Hankins et al., 2012).

Although there are many examples of acyl chain modification of lipid A, the mechanism by which changes in the number of these chains differentially affects TLR4 signaling has not been fully elucidated. However, the species-specific nature of the response has aided the development of this understanding. In the next section, we will review crystal structure and biochemical evidence that describes how the tetra-acyl precursor of lipid A, lipid IVA, is recognized by human and mouse TLR4-MD-2 complexes.

### How is lipid a with fewer than 6 acyl chains recognized?

The extracellular portion of TLR4, particularly in an 82-amino acid hypervariable middle region, is poorly conserved across species (Hajjar et al., 2002), which suggests there is increased selective pressure for this region of TLR4 to “cope” with variable ligand structure. Indeed, while hexa-acylated lipid A is a potent agonist for both mouse and human TLR4, the tetra-acylated precursor molecule generated during the course of the biogenesis of lipid A, lipid IVA (Figure 3D), induces species-specific TLR4 responses. Lipid IVA displays antagonist activity for TLR4 signaling in cells expressing human TLR4 and MD-2, but has agonist activity for mouse (Akashi et al., 2001) and horse (Walsh et al., 2008) TLR4-MD-2. The generation of inter-species chimeric TLR4 and MD-2 molecules which swap regions important for recognition of lipid IVA have greatly contributed to our understanding of how variation in lipid A is differentially recognized (Muroi and Tanamoto, 2006; Walsh et al., 2008).

The crystal structures of lipid IVA in complex with both human TLR4-MD-2 (Ohto et al., 2007) and mouse TLR4-MD-2 (Ohto et al., 2012a) have been described. In both structures, all four acyl chains sit inside the MD-2 binding pocket (Ohto et al., 2007). This implies a loss of the first dimerization interface, at which the acyl chain that sits outside of the pocket interacts with TLR4^*^. Furthermore, the size of the MD-2 binding pocket is unchanged regardless of whether it accommodates four- or six-chain lipid A; thus, in the presence of hexa-acyl lipid A, extra space is generated by pushing the diglucosamine backbone upwards, thereby contributing to the creation of the second dimerization interface between positively-charged residues in TLR4^*^ and negatively-charged phosphate groups on lipid A. This does not occur in the structure of lipid IVA and human MD-2 (Ohto et al., 2012a), and thus antagonistic lipid IVA occupies the human TLR4-MD-2 complex without triggering dimerization.

However, lipid IVA is an agonist for mouse and horse TLR4-MD-2 (Akashi et al., 2001; Walsh et al., 2008). Notably, unlike in the human complex, lipid IVA and lipid A have the same overall orientation in the mouse MD-2 binding pocket (Ohto et al., 2012a): the diglucosamine backbone of lipid IVA is shifted upward by about 4–7 Å, similar to lipid A. This was predicted by Meng et al. using molecular docking software (Meng et al., 2010b). The presence of a negatively-charged glutamate residue (E122) at the mouth of the MD-2 binding pocket repulses the negatively-charged phosphate group on lipid IVA, and pushes the backbone upward (Meng et al., 2010b). This model was confirmed by the crystal structure (Ohto et al., 2012a), which shows that indeed the first dimerization interface is preserved, as the C3–C7 atoms of the R2 acyl chain of lipid IVA are exposed to solvent and make van der Waals contacts with F438^*^ of TLR4^*^. Furthermore, the two phosphate groups, which are important for activation of TLR4-MD-2, make direct hydrogen bonds with both TLR4 and TLR4^*^. The 1-phosphate of lipid IVA was found to be surrounded by positively-charged residues, K341, K360 in TLR4, and K367^*^, R434^*^ in TLR4^*^, and R90 in MD-2. The 4′-phosphate was surrounded by K263, R266, K319, and R337 of TLR4. K367^*^ and R434^*^ were previously identified by site-directed mutagenesis to be important for lipid IVA-induced signaling through mouse TLR4 (Meng et al., 2010a,b), and are located at the second dimerization interface. Replacement of these with residues found at equivalent positions in human TLR4 (E369 and Q436) abolished the responsiveness of mouse TLR4-MD-2 to lipid IVA (Meng et al., 2010b).

Differences in the amino acid residues in MD-2 also contributed to the species-specific responses. In the antagonistic structure of human TLR4-MD-2 with lipid IVA, lipid IVA was confined more deeply in the hydrophobic cavity than in mouse. This is likely due to interaction of the phosphate group with K122 at the mouth of the human MD-2 binding pocket, which was thought to stabilize an antagonistic orientation of lipid IVA (Meng et al., 2010b), whereas the side chain of E122 in mouse MD-2 is oriented away from lipid IVA (Ohto et al., 2012a). Additionally, L125 of mouse MD-2 makes van der Waals contacts with N415^*^, M417^*^, L442^*^, and S443^*^ of TLR4^*^. L125 is not conserved in human MD-2 (K125). Thus, while changes in the number of acyl chains in lipid A affect its interaction with TLR4 and/or MD-2, the net effect on receptor dimerization and signaling through the TLR4-MD-2 complex is host-specific.

Crystal structure analysis of mouse TLR4-MD-2 in complex with lipid IVA showed that the receptor was dimerized (2:2:2), similar to lipid A, although in solution, mouse TLR4-MD-2 in complex with lipid IVA forms only monomers, while lipid A induced the formation of dimers (Ohto et al., 2012a). Here, the authors postulated that while both are agonists, lipid IVA is a weaker agonist than lipid A, and therefore that the interaction would not be strong enough to maintain dimer formation in solution (Ohto et al., 2012a).

The synthetic molecule Eritoran (Figure 3E) is derived from *Rhodobacter sphaeroides* LPS and was first investigated for its potential as an anti-sepsis therapeutic due to its antagonistic activity on TLR4 signaling in both mouse and human cells (Mullarkey et al., 2003; Visintin et al., 2005). Like lipid IVA, Eritoran also has only 4 acyl chains, all of which fit in the MD-2 binding pocket and take up 90% of its solvent-accessible space (Kim et al., 2007). Two of the acyl chains (R2 and R3) are fully extended in the pocket and two (R2′ and R3′) are bent in the middle. In this conformation, the diglucosamine backbone of Eritoran is fully exposed to solvent, and the two phosphate groups form ionic bonds with multiple positively-charged residues at the opening of the MD-2 pocket. Eritoran does not interact directly with TLR4 and does not induce TLR4-MD-2 complex dimerization, as measured by gel filtration chromatography (Kim et al., 2007).

Taken together, these data show that changes in the number of acyl chains on lipid A can affect both the first dimerization interface, involving the fatty acyl chain of lipid A and hydrophobic amino acid residues on TLR4^*^, as well as the second dimerization interface, involving the 1-phosphate of lipid A with positively-charged amino acid residues on TLR4^*^, due to changes in how lipid A is positioned inside MD-2. Furthermore, differences in the availability of charged amino acid residues in mouse and human MD-2 and TLR4/TLR4^*^ contribute to species-specific responses.

In the next section, we will review how the phosphate groups of lipid A at the second dimerization interface can be varied, as well as the effect of some of these modifications on the host immune response.

### Variations in lipid a phosphate groups and their effect on the host immune response

The importance of the phosphate groups on lipid A has been investigated using monophosphoryl lipid A (MPLA), a synthetic and less toxic derivative of lipid A from *S.* Typhimurium (Figure 3F). MPLA stimulation yielded low levels of the pro-inflammatory cytokine IL-6, but robust levels of IP-10, IFNβ, and MCP-1 in bone marrow-derived macrophages (Mata-Haro et al., 2007). This suggests that loss of the 1-phosphate group leads to normal activation of the TRIF pathway and poor activation of the MyD88 pathway (Mata-Haro et al., 2007). Thus, modification of lipid A phosphorylation may represent an important means of modulating the activation of TLR4 and the downstream immune response for therapeutic purposes. Interestingly, an earlier study also showed that MPLA induced low levels of interferon gamma, IL-12 p40 and p35, but robust levels of IL-10 in mouse macrophages cultures (Salkowski et al., 1997), perhaps pointing to an additional, as yet uncharacterized, role for lipid A modification in directing the downstream adaptive immune response.

Recent work has demonstrated that host-specific differences in TLR4 can affect responses to phosphorylation variants of lipid A. Human polymorphisms in TLR4 have been reported, including D299G and T399I (Arbour et al., 2000). D299G, in particular, has been associated with susceptibility to septic shock (Lorenz et al., 2002), airway hyporesponsiveness to inhaled endotoxin (Arbour et al., 2000), and defects in the recruitment of MyD88 and TRIF upon treatment with *E. coli* LPS (Figueroa et al., 2012). The D299G polymorphism causes a structural change at a site distal to LPS binding (Ohto et al., 2012b) and D299G TLR4 showed increased hyporesponsiveness to MPLA compared to *E. coli* lipid A (Yamakawa et al., 2013). Thus, human polymorphisms in TLR4 could have a significant impact on TLR4-mediated immunity against pathogens that express monophosphorylated species of lipid A.

Interestingly, lipid A phosphorylation is modified in several species, including *P. gingivalis*, where dephosphorylation of lipid A was linked to significantly poorer NFκB activation and additionally reduced affinity for antimicrobial peptides (Coats et al., 2009a,b). *Francisella novicida* has been shown to express a tetra-acylated (16 or 18C) lipid A, with just one phosphate group (1-phosphate), which induces no TLR4 response, agonist or antagonist, in either human or mouse macrophages (Hajjar et al., 2006). This finding suggests it is not recognized by TLR4, and the authors speculated that the effect might be upstream of TLR4/MD-2, perhaps a defect in recognition by LBP or CD14. The enzymes that catalyze the addition or removal of the phosphate groups of lipid A have been described in *Francisella tularensis* and *F. novicida*: LpxE and LpxF are integral inner membrane enzymes that dephosphorylate lipid A in the periplasmic leaflet of the inner membrane. LpxE removes the 1-phosphate group from penta- or hexa-acylated lipid A (Wang et al., 2004). LpxF removes the 4′-phosphate group from penta- or tetra-acylated lipid A (Wang et al., 2006).

Homologues of LpxE or LpxF are not known to naturally occur in *Salmonella*, but expression of these enzymes in *S.* Typhimurium led to the generation of mono-phosphate or non-phosphorylated lipid A mutants. LPS purified from these mutants induced reduced levels of the pro-inflammatory cytokines IL-6, IL-1β, and TNFα in macrophage cell lines, and orally-administered bacteria were attenuated for colonization in BALB/c mice, although sufficiently immunogenic to protect the host from challenge with wildtype *S.* Typhimurium (Kong et al., 2012). Thus, mono-phosphorylated (or non-phosphorylated) lipid A is less immunostimulatory for TLR4, but must non-etheless induce some protective immunity. LpxT is an enzyme that catalyzes the addition of a phosphate group to generate lipid A 1-diphosphate (Touze et al., 2008). In its absence, lipid A undergoes increased modification with phosphoethanolamine (Herrera et al., 2010), which would lead to increased resistance to antimicrobial peptides. The effect of the extra phosphate group on TLR4 activation has not been characterized, and its effect on the pathogenicity of the bacterium, or the host immune response to it, is unknown.

Several enzymes that modify lipid A via the addition of various chemical groups have been described. ArnT adds a 4-amino-4-deoxy-L-arabinose moiety to the 4′-phosphate group (Trent et al., 2001b), which increases resistance to polymyxin B and other CAMPs in *S.* Typhimurium (Gunn et al., 1998). The neutral aminoarabinose moiety is thought to reduce the negative charge on the bacterial membrane and thereby reduce the binding affinity for cationic peptides and killing. The Arn operon has been found in *E. coli, S.* Typhimurium, and *P. aeruginosa* (Trent et al., 2001b). The effect of the aminoarabinose moiety on TLR4 signaling is unclear, as chemical removal of this group from lipid A from *P. aeruginosa* does not yield any significant change in TLR4 activity (Hajjar et al., 2002). However, disruption of aminoarabinose addition did negatively affect PagL deacylase activity (Kawasaki et al., 2005a), suggesting a possible indirect effect of this modification on TLR4 activation.

PmrC catalyzes the addition of a phosphoethanolamine group to 1- or 4′-phosphates (Lee et al., 2004). It is induced by PmrA, and is linked to resistance to CAMPs in *S. enterica* (Lee et al., 2004). Blocking phosphoethanolamine addition in *Neisseria gonorrhoeae* (by inactivation of LptA, the enzyme catalyzing the addition of phosphoethanolamine in *N. gonorrhoeae*) was found to increase susceptibility to polymyxin B and complement-mediated killing (Lewis et al., 2009). LpxO (PagQ) incorporates a hydroxyl group into 2-hydroxymyristate lipid A and is also under the control of PhoP/Q (Gibbons et al., 2000; Raetz, 2001). The effects of these enzymatic modifications on signaling through TLR4 have not been described.

Glucosamine modification of lipid A (Figure 3G) from *B. pertussis* strain BP338, a Tohama I derivative, by an ArnT ortholog is critical for its agonist activity in cells expressing human TLR4, although it is dispensable for cells expressing mouse TLR4 (Marr et al., 2010a). Mutants lacking glucosamine modification induced significantly decreased levels of the pro-inflammatory cytokines TNFα, IL-6, and IL-1β, as well as the TRIF-dependent chemokines IP-10 and RANTES, in a human macrophage cell line, and showed decreased activation of NFκB in reporter assays. A different lipid A species from *B. pertussis* strain 18-323 is an antagonist in humans and a poor agonist in mice (Marr et al., 2010b). This lipid A is also not glucosamine modified, and furthermore has a shorter (10 or 12C instead of 14C) R3′ acyl chain. These data show that the addition of positively-charged chemical groups can also contribute to differential signaling. Whether the differences in acyl chain length in *B. pertussis* affect TLR4 signaling through alteration of the first dimerization interface (acyl chain and TLR4^*^) or by altering the position of lipid A, and thus the interaction of the 1-phosphate group with positively-charged residues in TLR4^*^ at the second dimerization interface, is unclear.

## Conclusion

Although much work has gone into characterizing various lipid A species and the enzymes involved in their modification, the mechanism by which these changes are recognized by TLR4, one of the very first steps of the host immune response to the pathogen, has been limited to a few structures. The recent crystal structure analyses of human and mouse TLR4/MD-2 receptors in complex with lipid A, lipid IVA, and Eritoran have provided insight into how differences in acyl chain numbers can affect the position of lipid A in the MD-2 binding pocket, and how lipid A interacts with TLR4 and TLR4^*^ to induce receptor dimerization. Many more questions remain to be answered; for example, how are lipid A molecules with shorter acyl chains coordinated? How do modifications to phosphate groups affect TLR4 crosslinking? It has been proposed that the addition of positively-charged chemical moieties onto the phosphate groups functions to reduce the negative charge of the outer membrane and thereby decreases susceptibility to CAMPs, but it is conceivable that positively-charged groups might also affect how TLR4 recognizes lipid A, and this has been demonstrated in the case of glucosamine modification of *B. pertussis* lipid A (Marr et al., 2010a).

The relative contribution of each dimerization interface involved in TLR4 recognition of hexa-acyl lipid A is unclear, although mutational analysis has shown that both positively-charged residues in TLR4^*^, as well as the F440 residue in TLR4^*^ that interacts with the R2 acyl chain of lipid A, are required for optimal activation of NFκB (Resman et al., 2009; Meng et al., 2011). Interestingly, loss of the 1-phosphate group on MPLA results in preferential signaling through the TRIF, but not MyD88-dependent pathway. The mechanism for how this might occur is unclear. Gangloff has proposed a model whereby in acidic endosomes (pH 5.5), the introduction of positive charges on histidine residues in the extracellular domain of TLR4 leads to the formation of an “alternative dimerization mode,” one that would allow MPLA to signal in endosomes and activate the TRIF pathway (Gangloff, 2012).

Clearly, much can still be gleaned from a more comprehensive understanding of this interaction, especially as effects of phosphate modification on downstream immune responses (dendritic cell maturation, cytokine production, innate cell recruitment, bacterial clearance) have been reported (Mata-Haro et al., 2007), yet there is still little known about how changes in lipid A engagement of TLR4, and the subsequent dimerization of the TLR4-MD-2 complex, can lead to such diverse responses. The potential importance of this understanding is highlighted by a study that showed that various Gram-negative pathogens can differentially stimulate the production of MyD88-dependent and TRIF-dependent cytokines and chemokines (Zughaier et al., 2005). Hexa-acylated LPS from *E. coli* and *V. cholerae* stimulated robust TNFα, which could be inhibited by dominant negative MyD88, but lower levels of nitrite or IP-10, typical indicators of TRIF-pathway activation, compared to LPS from *S*. Typhimurium or *S*. Minnesota. On the other hand, *N. meningitidis*, which expresses a hexa-acylated LOS, produced robust levels of TNFα, IP-10, and nitrite (Zughaier et al., 2005). A more complete understanding of how lipid A variants affect TLR4 signaling is expected to aid vaccine design, both for improving adjuvanticity and for minimizing undesirable inflammatory responses, as well as potentially providing insight into pathogen-specific immunomodulation strategies. Interestingly, TLR4 has also been shown to mediate LPS-induced autophagy in human and mouse macrophages (Xu et al., 2007; Shi and Kehrl, 2008) and repeated exposure to LPS induces tolerance in macrophages, monocytes, as well as dendritic cells (Biswas and Lopez-Collazo, 2009). Both of these LPS-induced responses have significant implications for the host-pathogen interaction, but the effect of lipid A modification is unknown.

Although this review focused on monomeric LPS (lipid A) interactions with TLR4, the contribution of LPS aggregates on the host response, perhaps upstream or independent of LBP and CD14 (Sasaki and White, 2008), may also be an important consideration. In addition, previous work has suggested that some LPS variants have a decreased ability to bind CD14 or LBP, upstream of TLR4 (Cunningham et al., 1996; Neumeister et al., 1998). The crystal structure of CD14 suggests that lipid A may bind on a large hydrophobic face in the N-terminal region of the molecule (Kim et al., 2005); however, it is at present still unclear how lipid A modification may affect its recognition and binding to CD14 or LBP.

Intriguingly, the ability of host TLR4-MD-2 complexes to differentially respond to lipid A variants is, at least to some degree, species-specific (Akashi et al., 2001; Hajjar et al., 2002, 2012; Walsh et al., 2008; Marr et al., 2010a,b). Further analysis of how different lipid A structures from Gram-negative pathogens are recognized by host TLR4-MD-2 receptors is expected to provide insight into how pathogens have evolved to fine tune the very first step in their recognition by, and interaction with, host innate receptors, and additionally how host receptors may have evolved to respond to these pathogens.

### Conflict of interest statement

The authors declare that the research was conducted in the absence of any commercial or financial relationships that could be construed as a potential conflict of interest.
